# Increased frequency of *CHEK2* germline pathogenic variants among individuals with dermatofibrosarcoma protuberans

**DOI:** 10.1016/j.gimo.2024.101895

**Published:** 2024-09-28

**Authors:** Michael R. Sargen, Jung Kim, Jeremy S. Haley, Hayley P. Barker, Piyushkumar A. Mundra, Mandy L. Ballinger, David M. Thomas, David J. Carey, Alisa M. Goldstein, Douglas R. Stewart

**Affiliations:** 1 Division of Cancer Epidemiology and Genetics, National Cancer Institute, National Institutes of Health, Rockville, MD; 2 Department of Genomic Health, Geisinger Clinic, Geisinger Health System, Danville, PA; 3 Garvan Institute of Medical Research, Sydney, NSW, Australia; 4 St Vincent’s Clinical School, University of New South Wales, Sydney, NSW, Australia; 5 School of Biomedical Sciences, University of New South Wales, Sydney, NSW, Australia; 6 Centre for Molecular Oncology, University of New South Wales, Sydney, NSW, Australia

**Keywords:** Cancer predisposition, *CHEK2*, Dermatofibrosarcoma protuberans, Sarcoma, Skin cancer

## Abstract

**Purpose:**

To identify candidate susceptibility genes for dermatofibrosarcoma protuberans (DFSP).

**Methods:**

All individuals with DFSP from the International Sarcoma Kindred Study (*n* = 3767 individuals with sarcoma diagnoses from Australia, Europe, New Zealand, and United States) and cohorts that were not ascertained based on sarcoma status or other phenotypes (Geisinger MyCode, *n* = 170,503 individuals, United States; UK Biobank, *n* = 469,789 individuals, United Kingdom) were evaluated for germline pathogenic or likely pathogenic (P/LP) variants in 156 cancer genes.

**Results:**

There were 92 unrelated individuals with DFSP across the 3 cohorts. The mean age at diagnosis (standard deviation) in the International Sarcoma Kindred Study, Geisinger, and UK Biobank was 40.8 (14.5), 50.3 (9.4), and 49.4 (13.2) years, respectively. Germline P/LP variants were most common in the *CHEK2* gene (4/92 [4.3%]). *CHEK2-*related cases were often associated with early onset disease (age at diagnosis: 30-39 years) and were observed in all 3 cohorts. Among 640,292 individuals in Geisinger and UK Biobank who were not ascertained based on phenotype, there was a significantly increased frequency of *CHEK2* P/LP variants among individuals with DFSP (*n* = 3/65 [4.6%]) compared to those without (*n* = 6388/640,227 [1.0%]) (Fisher exact, *P* = .03). Additional genes with P/LP variation (1 case for each gene) included *ACD, ERCC5, ERCC1, DOCK8, GBA1, ATM, MUTYH, TP53, RECQL4,* and *COL7A1*.

**Conclusion:**

This study of multiple cohorts identifies *CHEK2* as a candidate susceptibility gene for DFSP. Additional epidemiologic and functional studies are needed to further characterize this potential gene-tumor relationship.

## Introduction

Dermatofibrosarcoma protuberans (DFSP) is a rare cutaneous sarcoma that often mimics common benign skin growths, such as keloid scars or cutaneous cysts, which can delay diagnosis.^1^ A consequence of this delay is that large excisions are typically needed for DFSP because of extensive tumor growth before disease recognition.^2^

Reports of familial DFSP suggest that hereditary factors may contribute to some cases.^3^^,^^4^ Multiple DFSP tumors have been reported in individuals with autosomal recessive *ADA*-deficient severe combined immune deficiency, although it is unknown if a single pathogenic variant of *ADA* or variation in other genes increases risk for this cancer.^5^ Germline analyses of DFSP are lacking and identifying hereditary factors associated with this sarcoma could improve disease recognition and our understanding of disease etiology. To address this knowledge gap, we evaluated the frequency of germline pathogenic (P) or likely pathogenic (LP) variants in 156 cancer susceptibility genes among individuals with DFSP in 3 large cohorts.

## Materials and Methods

### Cohorts

We used *International Statistical Classification of Diseases and Related Health Problems, Tenth Revision (ICD-10)* histology codes 8832 and 8833 to identify all DFSP cases in the Geisinger MyCode^6^ (*n* = 170,503 individuals in Geisinger Health System, Pennsylvania, United States) and UK Biobank^7^ (*n* = 469,789 individuals in the United Kingdom) cohorts, which link germline exome or genome and longitudinal electronic health records data. Individuals were eligible to participate in these studies regardless of their medical history (healthy volunteers or individuals with known disease), an approach that minimizes ascertainment biases based on known phenotypes.^8^ We also evaluated individuals with DFSP in the International Sarcoma Kindred Study (ISKS; *n* = 3767 individuals), which recruited individuals with sarcoma diagnoses regardless of their family history from sarcoma clinics in Australia, Europe, New Zealand, and the United States.^9^ Germline genome sequencing was performed for all individuals in the ISKS cohort.

### Variant classification

Among individuals with DFSP, we evaluated for P/LP variants in 156 cancer susceptibility genes (Supplemental Table 1) described in prior studies, including genes identified by Ballinger et al^9^ that predispose to non-DFSP sarcomas.^9^^,^^10^ We initially filtered the gene data for rare variants with an allele frequency ≤.005 in the Genome Aggregation Database (gnomAD v2.1.1).^11^ Variants were denoted as P or LP if they were classified as such in ClinVar (as of 10/01/2023),^12^ a public database of reports of the relationships among human genetic variations and clinical phenotypes. Variants with conflicting interpretations of pathogenicity in ClinVar were classified as LP if the majority of ClinVar entries for the variant were P or LP. For variants of uncertain significance (VUS) or those not found in ClinVar, they were upgraded to P or LP if they were predicted to have this effect based on InterVar,^13^ a bioinformatics tool that interprets variants based on 2015 American College of Medical Genetics and Genomics and the Association for Molecular Pathology guidelines. Differences in variant frequency based on DFSP status (with vs without DFSP) were evaluated using Fisher exact tests if the variant was present in ≥2 individuals with DFSP. Statistical tests were performed in Stata 17.0.

## Results

In total, there were 92 unrelated individuals with DFSP across the 3 cohorts (Table 1). The mean age at diagnosis (standard deviation [SD]) in ISKS, Geisinger, and UK Biobank was 40.8 (14.5), 50.3 (9.4), and 49.4 (13.2) years, respectively.

**Table 1:** Clinical characteristics of cohorts

	Individuals, No. (%)		
Characteristic	Geisinger MyCode	UK Biobank	ISKS
No. of individuals (%)	*N* = 170,503 (100)	*N* = 469,789 (100)	*N* = 3767 (100)
Female	103,365 (60.6)	254,626 (54.2)	1829 (48.6)
Male	67,138 (39.4)	215,163 (45.8)	1938 (51.4)
Current age, years			
Mean (SD)	58.9 (19.1)	70.0 (8.0)	45.5 (37.6)
Median age	60.9	71	47
Race and ethnicity^a^			
Black or African	3695 (2.2)	17,188 (3.7)	20 (0.5)
White	164,078 (96.2)	445,337 (94.8)	1218 (32.3)
Unknown or Other	2730 (1.6)	7264 (1.5)	2529 (67.1)^b^

No. of individuals with dermatofibrosarcoma protuberans	*n* = 20 (100)	*n* = 45 (100)	*n* = 27 (100)

Female	11 (55)	27 (60)	12 (44)
Male	9 (45)	18 (40)	15 (56)
Age at diagnosis, years			
Mean (SD)	50.3 (9.4)	49.4 (13.2)	40.8 (14.5)
Median	48.5	50.5	40
Race and ethnicity^a^			
White	19 (95)	42 (93)	12 (44)
Black or African American	1 (5)	3 (6.7)	0
Unknown	0	0	15 (56)
Body site			
Head and neck	1 (5)	7 (16)	3 (11)
Lower Extremities	3 (15)	2 (4.4)	11 (41)
Trunk including genitalia	11 (55)	22 (49)	6 (22)
Upper Extremities	5 (25)	13 (29)	7 (26)
Not Specified	0	1 (2.2)	0

ISKS, International Sarcoma Kindred Study; SD, standard deviation.

a Sex and race were self-reported. UK Biobank participants were classified as White for race and ethnicity if they had a skin color code (variable p1717) for very fair, fair, light olive, or dark olive and were classified as Black or African American if they had a skin color code for brown or black.

b All individuals had unknown race and ethnicity.

Of the 92 DFSP cases, there were 4 (*n* = 4/92 [4.3%]) individuals with P/LP variants of *CHEK2* (HUGO Gene Nomenclature Committee [HGNC] ID: 16627; NM_007194.4, NP_009125.1): 3 individuals with c.470T>C p.(Ile157Thr) and 1 individual with c.1100del p.(Thr367MetfsTer15) (Table 2). Because of the multiple cases associated with germline P/LP variants of *CHEK2*, we searched for VUS in this gene that were classified as P/LP by at least 1 lab in ClinVar among individuals with DFSP. This search found one *CHEK2* VUS (c.190G>A p.(Glu64Lys); ClinVar Variation ID: 128068) satisfying this criteria in a DFSP patient from the ISKS cohort.

**Table 2:** Germline pathogenic or likely pathogenic variants among individuals with dermatofibrosarcoma protuberans

							Phenotype Ascertainment	Non-Phenotype Ascertainment				
							ISKS	Geisinger MyCode		UK Biobank		Geisinger MyCode and UK Biobank
							With DFSP	With DFSP	Without DFSP	With DFSP	Without DFSP	***P***^a^
No. of individuals (%)							27 (100)	20 (100)	170,483 (100)	45 (100)	469,744 (100)	
Gene^b^	CHR	POS	REF	ALT	HGVS c.	HGVS p.						
*CHEK2*							1 (3.7)	2 (10)	3153 (1.8)^c^	1 (2.2)	3235 (0.7)^c^	.03
	22	28725099	A	G	c.470T>C	p.(Ile157Thr)	1 (3.7)	2 (10)	1824 (1.1)	0	586 (0.1)	
	22	28695868	AG	A	c.1100del	p.(Thr367MetfsTer15)	0	0	693 (0.4)	1 (2.2)	1319 (0.3)	
*ACD*	16	67659992	CG	C	c.152del	p.(Thr51SerfsTer21)	0	1 (5)	0	0	4 (0.0009)	
*ERCC5*	13	102868199	G	A	c.2620G>A	p.(Ala874Thr)	0	1 (5)	67 (0.04)	0	49 (0.01)	
*ERCC1*	19	45414036	T	C	c.703-2A>G	p.?	0	1 (5)	0	0	9 (0.002)	
*DOCK8*	9	312071	C	T	c.646C>T	p.(Gln216Ter)	0	1 (5)	0	0	1 (0.0002)	
*GBA1*	1	155238174	C	T	c.721G>A	p.(Gly241Arg)	0	1 (5)	0	0	18 (0.004)	
*ATM*	11	108293324	G	GT	c.4625dup	p.(Leu1542PhefsTer8)	0	0	0	1 (2.2)	5 (0.00001)	
*MUTYH*	1	45331556	C	T	c.1103G>A	p.(Gly368Asp)	0	0	1907 (1.1)	1 (2.2)	5123 (1.1)	
*TP53*	17	7675994	C	T	c.375G>A	p.(Thr125=)	1 (3.7)	0	0	0	0	
*RECQL4*	8	144514982	CA	C	c.1573del	p.(Cys525AlafsTer33)	1 (3.7)	0	0	0	0	
*COL7A1*	3	48590585	C	T	c.1781-1G>A	p.?	1 (3.7)	0	0	0	0	

*ALT*, alternative; *CHR*, chromosome; *DFSP*, dermatofibrosarcoma protuberans; *HGVS*, Human Genome Variation Society; *ISKS*, International Sarcoma Kindred Study; *POS*, position; *REF*, reference.

a Fisher exact test assessing variant frequency based on DFSP status (with vs without DFSP) was performed for variants detected in ≥2 individuals with DFSP.

b Variants were denoted as pathogenic (P) or likely pathogenic (LP) if they were so classified in ClinVar (as of 10/01/2023). Variants with conflicting interpretations of pathogenicity in ClinVar were classified as LP if the majority of ClinVar entries for the variant were P or LP. For variants of uncertain significance (VUS) in ClinVar, they were upgraded to P or LP if they were predicted to have this effect based on InterVar. Variant coordinates are based on Genome Reference Consortium Human Build 38 (GRCh38).

c This group includes all individuals with *CHEK2* germline P/LP variants, including variants (not shown) that were detected only in people without DFSP.

Two DFSP cases associated with germline P/LP variants of *CHEK2* and 1 case associated with the *CHEK2* VUS p.(Glu64Lys) were diagnosed between ages 30 to 39 (Supplemental Table 2). An individual with DFSP and germline LP variant p.(Ile157Thr) had multiple biopsy confirmed dysplastic nevi, whereas another individual with this variant was diagnosed with cutaneous melanoma. Prostate cancer was also observed in a DFSP case associated with *CHEK2* germline VUS p.(Glu64Lys) (Supplemental Table 2). Four *CHEK2*-related DFSP cases occurred in White individuals and 1 case occurred in a person with unknown race and ethnicity. Most *CHEK2*-related DFSP cases occurred in men (*n* = 4/5 [80%]) (Supplemental Table 2).

Among 640,292 individuals in Geisinger and UK Biobank who were not ascertained based on phenotype, there was a significantly increased frequency of *CHEK2* germline P/LP variants among individuals with DFSP (*n* = 3/65 [4.6%]) compared with those without this cancer (*n* = 6388/640,227 [1.0%]) (Fisher exact, *P* = .03) (Table 2). In Geisinger, we also observed an increased frequency (*P* = .02) for the most common variant, p.(Ile157Thr), among individuals with DFSP (*n* = 2/20 [10%]) compared with those without this cancer (*n* = 1824/170,483 [1.1%]). Among individuals with DFSP in the 3 cohorts, germline P/LP variants were also identified in the following genes (1 case per gene): *ACD* (HGNC ID: 25070), *ERCC5* (HGNC ID: 3437), *ERCC1* (HGNC ID: 3433), *DOCK8* (HGNC ID: 19191), *GBA1* (HGNC ID: 4177), *ATM* (HGNC ID: 795), *MUTYH* (HGNC ID: 7527), *TP53* (HGNC ID: 11998), *RECQL4* (HGNC ID: 9949), and *COL7A1* (HGNC ID: 2214) (Table 2). *ADA* (HGNC ID: 186) germline P/LP variants were not detected in individuals with DFSP. Variant classifications and their supporting evidence are available in Supplemental Table 3.

## Discussion

In this germline analysis of 3 large cohorts with linked exome or genome and electronic health records data, we identified an increased frequency of *CHEK2* germline P/LP variants among individuals with DFSP, including 3 individuals with missense variant p.(Ile157Thr) and 1 individual with truncating variant p.(Thr367MetfsTer15). These variants are the 2 most common European founder variants in *CHEK2*.

*CHEK2* is a low-penetrance cancer susceptibility gene that increases risk for breast, colorectal, and prostate cancer.^14^^,^^15^ Several studies have also identified an association between *CHEK2* variant p.(Thr367MetfsTer15) and increased melanoma risk.^14^^,^^16^ In our study, there was 1 individual with *CHEK2*-related DFSP who developed cutaneous melanoma, although the variant was p.(Ile157Thr) instead of p.(Thr367MetfsTer15). Another individual with DFSP and p.(Ile157Thr) had multiple dysplastic nevi but no reported melanomas.

We also identified another individual with DFSP who had *CHEK2* p.(Thr367MetfsTer15), and this specific variant has previously been reported in an individual with early onset DFSP (age at diagnosis 17 years).^17^ Interestingly, *CHEK2*-related DFSP cases in this study often occurred in the third decade of life, which is considerably younger than the mean age at diagnosis for this cancer in Geisinger and UK Biobank. The *CHEK2* variant p.(Thr367MetfsTer15) has also been associated with an increased risk for sarcomas in a large Danish study, although this association was not significant after adjustment for multiple comparisons.^18^

The p.(Ile157Thr) variant was the most common *CHEK2* P/LP variant among individuals with DFSP (*n* = 3 cases).^15^ This variant has been associated with an increased risk for breast carcinogenesis, although risk for this missense variant appears to be lower compared with frameshift variants according to National Comprehensive Cancer Network guidelines for genetic risk assessment for breast cancer.^19^ However, it remains unsettled whether the p.(Ile157Thr) variant predisposes to breast cancer because of its frequency in certain populations, which approaches 2% in people of European ancestry (gnomAD v2.1.1). Additionally, functional analyses of this variant have shown mixed results with 1 study suggesting that the variant impairs protein function^20^ and a second study suggesting that the variant is not damaging.^21^

Despite the mixed results of functional analyses, most ClinVar (Variation ID: 5591) entries for p.(Ile157Thr) are P/LP: P (*n* = 6 entries), LP (*n* = 13 entries), P, low penetrance (*n* = 1 entry), established risk allele (*n* = 1 entry), and uncertain significance (*n* = 9 entries). This variant is also predicted to be LP based on InterVar. Additionally, the frequency of this variant in the Geisinger cohort was increased (*P* = .02) among individuals with DFSP compared with individuals without this cancer, suggesting that this variant could be a risk factor for DFSP.

Early tumor onset, another feature of germline variants that predispose to cancer, was also observed in a Geisinger patient with the p.(Ile157Thr) variant who developed DFSP between ages 30 to 39 years, considerably younger than the mean age at diagnosis in the Geisinger cohort (50.3 years). Together, our epidemiologic data along with ClinVar and InterVar predictions of pathogenicity suggest that p.(Ile157Thr) could be a susceptibility variant for DFSP. Confirmation of this association in diverse populations would further support this hypothesis.

One individual in the ISKS cohort with DFSP had the *CHEK2* germline variant p.(Glu64Lys). There remains uncertainty about whether this variant predisposes to cancer. Although 10 entries in ClinVar classify this variant as LP, the majority of entries classify this variant as uncertain significance (*n* = 15 entries) (variation ID: 128068). Additionally, this variant is also classified as a VUS based on InterVar criteria. Although 2 functional studies have found that the p.(Glu64Lys) variant has an intermediate impact on protein function, the results were indeterminate as to whether the altered gene product would impair CHK2 activity enough to cause disease.^20^^,^^21^

In this study, our patient in the ISKS cohort with the *CHEK2* germline variant p.(Glu64Lys) developed DFSP at a relatively young age (30s) compared with the mean age of diagnosis in ISKS (40.8 years), Geisinger (50.3 years), and UK Biobank (49.4 years). There are also epidemiologic data to suggest that this variant could be damaging and contributing to cancer. For example, the p.(Glu64Lys) variant is enriched in breast cancer patients compared with controls in data sets from Australia, Czech Republic, Germany, Spain, Italy, and the United States.^20^ Additionally, our patient with DFSP and the *CHEK2* germline variant p.(Glu64Lys) developed prostate cancer, another *CHEK2*-associated cancer, suggesting that this genetic alteration could be damaging to CHK2 activity and predispose to cancer formation.^14^

The CHK2 protein is a critical component of DNA double-strand break repair, which protects cells from accumulating translocations and other damaging structural aberrations.^22^ DFSP arises from a translocation of chromosomes 17 (*COL1A1*, HGNC ID: 2197) and 22 (*PDGFB*, HGNC ID: 8800). Therefore, loss of *CHEK2* could potentially increase the likelihood of developing this tumorigenic translocation.

Our study has several important limitations that could impact the interpretation of results. Although we did not identify germline P/LP variants of *ADA*, the possibility of this gene contributing to DFSP cannot be entirely excluded given the limited number of cases available for evaluation. It is also possible that other genes not evaluated in this study could be contributing to some DFSP cases. Underlying population frequencies of *CHEK2* variants could also potentially be impacting the observed findings. Therefore, it is possible that the observed findings may not extend beyond the populations analyzed. Although germline *CHEK2* P/LP variants were more common in individuals with DFSP, a large number of individuals with *CHEK2* P/LP variants in Geisinger and UK Biobank did not develop DFSP suggesting that *CHEK2*, if contributing to the formation of this cancer, is a low-risk susceptibility gene that acts in combination with other factors to increase risk. Many susceptibility genes, including *CHEK2*, often exhibit loss of heterozygosity within tumors.^23^ Therefore, future studies should evaluate for loss of *CHEK2* heterozygosity in DFSP to further assess the role of this gene in tumor formation. Additional epidemiologic and functional studies are also needed to determine if the 10 non-*CHEK2* genes with P/LP variants contribute to DFSP. We also did not adjust for multiple comparisons in this exploratory analysis. However, the observation of *CHEK2* P/LP variants among individuals with DFSP in multiple cohorts implicates *CHEK2* as a candidate susceptibility gene for DFSP that should be investigated further in diverse cohorts. We also analyzed 2 large genomic databases (Geisinger and UK Biobank) that did not enroll based on phenotype, which minimized potential ascertainment bias, an additional strength of the study.

In conclusion, the study findings implicate *CHEK2* as a candidate susceptibility gene for DFSP and provide a rationale for future epidemiologic and functional studies to evaluate this gene-tumor relationship more completely. Moreover, confirmation of this sarcoma as part of the *CHEK2* cancer spectrum could inform genetic testing recommendations for patients with DFSP.

## Data Availability

The data supporting the findings of this article are reported in the main text, figures, and tables. Data to reproduce the results are available to qualified academic noncommercial researchers under a data access agreement.

## Conflict of Interest

The authors declare no conflicts of interest.

## Supplemental Material

Supplemental Table 1 Cancer Susceptibility Genes Evaluated Among Individuals with Dermatofibrosarcoma Protuberans. Supplemental Table 2. Benign and Malignant Tumor Diagnoses Among Individuals with *CHEK2*-related Dermatofibrosarcoma Protuberans. Supplemental Table 3. Germline Variants Predicted to be Damaging to Protein Function or Expression Among Individuals with Dermatofibrosarcoma Protuberans.
