# Supplementary material for: Increased frequency of CHEK2 germline pathogenic variants among individuals with dermatofibrosarcoma protuberans
Source: Genet Med Open. 2024 Sep 28;2:101895. doi: 10.1016/j.gimo.2024.101895 (PMC11613564; doi:10.1016/j.gimo.2024.101895)
Supplement: Supplemental Table 1 — Cancer Susceptibility Genes Evaluated Among Individuals with Dermatofibrosarcoma Protuberans. Supplemental Table 2. Benign and Malignant Tumor Diagnoses Among Individuals with CHEK2-related Dermatofibrosarcoma Protuberans. Supplemental Table 3. Germline Variants Predicted to be Damaging to Protein Function or Expression Among Individuals with Dermatofibrosarcoma Protuberans. [file mmc1.pdf]

SUPPLEMENTARY TABLES

Supplementary Table 1. Cancer Susceptibility Genes Evaluated Among Individuals with Dermatofibrosarcoma Protuberans

Supplementary Table 2. Benign and Malignant Tumor Diagnoses Among Individuals with *CHEK2*-related Dermatofibrosarcoma Protuberans

Supplementary Table 3. Germline Variants Predicted to be Damaging to Protein Function or Expression Among Individuals with Dermatofibrosarcoma Protuberans

Supplementary Table 1. Cancer Susceptibility Genes Evaluated Among Individuals with Dermatofibrosarcoma Protuberans

ABCB11	*CEBPA*	*FAH*	*MEN1*	*PTEN*	*SMARCA4*	*VHL*
ACD	*CEP63*	*FANCA*	*MET*	*PTPN11*	*SMARCAL1*	*WAS*
ADA	*CEP72*	*FANCC*	*MLH1*	*RAD51C*	*SMARCB1*	*WRN*
ALK	*CEP89*	*FANCG*	*MSH2*	*RAD51D*	*SMARCE1*	*WT1*
APC	*CHEK2*	*FBXW7*	*MSH6*	*RAF1*	*SOS1*	*XPA*
ATM	*COL7A1*	*FGFR4*	*MTAP*	*RAP1A*	*SRY*	*XPC*
AXIN2	*CTNNB1*	*FH*	*MUTYH*	*RAP1B*	*SSNA1*	
BAP1	*CYLD*	*FLCN*	*NBN*	*RB1*	*STAG3*	
BCOR	*DDB2*	*GATA2*	*NF1*	*RECQL4*	*STAT3*	
BLM	*DICER1*	*GBA1*	*NF2*	*RET*	*STK11*	
BMPR1A	*DIS3L2*	*GJB2*	*NRAS*	*RHBDF2*	*SUFU*	
BRAF	*DKC1*	*GPC3*	*PALB2*	*RIT1*	*TERF1*	
BRCA1	*DOCK8*	*HAUS4*	*PAX5*	*RMRP*	*TERF2*	
BRCA2	*EGFR*	*HAUS5*	*PCM1*	*RUNX1*	*TERF2IP*	
BRIP1	*ELANE*	*HFE*	*PDGFRA*	*SBDS*	*TERT*	
BUB1B	*EPCAM*	*HGF*	*PHOX2B*	*SDHA*	*TGFBR1*	
CBL	*ERCC1*	*HMBS*	*PIK3CA*	*SDHAF2*	*TIMELESS*	
CDC73	*ERCC2*	*HRAS*	*PMS2*	*SDHB*	*TINF2*	
CDH1	*ERCC3*	*ITK*	*POLD1*	*SDHC*	*TMEM127*	
CDH1	*ERCC4*	*KIT*	*POLE*	*SDHD*	*TNFRSF6 (FAS)*	
CDK13	*ERCC5*	*KRAS*	*POLH*	*SERPINA1*	*TP53*	
CDK4	*ERCC6*	*LZTR1*	*POT1*	*SH2D1A*	*TRIM37*	
CDKN1B	*ERCC8*	*MAP2K1*	*PRKAR1A*	*SHOC2*	*TSC1*	
CDKN1C	*EXT1*	*MAP2K2*	*PRSS1*	*SLC25A13*	*TSC2*	
CDKN2A	*EXT2*	*MAX*	*PTCH1*	*SMAD4*	*UROD*	

Supplementary Table 2. Benign and Malignant Tumor Diagnoses Among Individuals with *CHEK2*-related Dermatofibrosarcoma Protuberans

Case	*CHEK2* variant[a]	Sex[b]	Race and ethnicity[b]	Benign tumors (age at diagnosis, y)	Cancers (age at diagnosis, y)
**Geisinger**					
Case 1	c.470T>C p.(Ile157Thr)	Female	White	Congenital nevus of right forearm (20-29); multiple histologically confirmed dysplastic nevi	DFSP (30-39)
Case 2	c.470T>C p.(Ile157Thr)	Male	White	None	DFSP (40-49); malignant melanoma of lower limb including hip (50-59)
**UK Biobank**					
Case 3	c.1100del p.(Thr367MetfsTer15)	Male	White	Benign neoplasm of colon, unspecified site (60-69)	DFSP (30-39)
**ISKS**					
Case 4	c.470T>C p.(Ile157Thr)	Male	Unknown	None	DFSP (60-69)
Case 5	c.190G>A p.(Glu64Lys)	Male	White	Multiple lipomas (30-39)	DFSP (30-39); prostate cancer (50-59)

Abbreviations: International Sarcoma Kindred Study (ISKS), Dermatofibrosarcoma Protuberans (DFSP)

[a] Variant annotation to cDNA and protein used the following transcripts: NM_007194.4 and NP_009125.1.

[b] Self-reported data.

Supplementary Table 3. Germline Variants Predicted to be Damaging to Protein Function or Expression Among Individuals with Dermatofibrosarcoma Protuberans

Gene	CHR	POS	REF	ALT	HGVS c.	HGVS p.	ClinVar Variation ID	Classification[a]
CHEK2								
	22	28725099	A	G	c.470T>C	p.(Ile157Thr)	5591	Likely Pathogenic
	22	28695868	AG	A	c.1100del	p.(Thr367MetfsTer15)	128042	Pathogenic
ACD	16	67659992	CG	C	c.152del	p.(Thr51SerfsTer21)	None	Likely Pathogenic[b]
ERCC5	13	102868199	G	A	c.2620G>A	p.(Ala874Thr)	16577	Likely Pathogenic[c]
ERCC1	19	45414036	T	C	c.703-2A>G	p.?	1208249	Likely pathogenic
DOCK8	9	312071	C	T	c.646C>T	p.(Gln216Ter)	None	Likely Pathogenic[b]
GBA1	1	155238174	C	T	c.721G>A	p.(Gly241Arg)	93459	Likely Pathogenic
ATM	11	108293324	G	GT	c.4625dup	p.(Leu1542PhefsTer8)	181875	Likely Pathogenic
MUTYH	1	45331556	C	T	c.1103G>A	p.(Gly368Asp)	5294	Likely pathogenic
TP53	17	7675994	C	T	c.375G>A	p.(Thr125=)	177825	Pathogenic
RECQL4	8	144514982	CA	C	c.1573del	p.(Cys525AlafsTer33)	6066	Pathogenic
COL7A1	3	48590585	C	T	c.1781-1G>A	p.?	None	Pathogenic[d]

Abbreviations: Chromosome (CHR), Position (POS), Reference (REF), Alternative (ALT), Human Genome Variation Society (HGVS)

[a] Variants were denoted as pathogenic (P) or likely pathogenic (LP) if they were so classified in ClinVar (as of 10/01/2023). Variants with conflicting interpretations of pathogenicity in ClinVar were classified as LP if the majority of ClinVar entries for the variant were P or LP. For variants of uncertain significance (VUS) in ClinVar, they were upgraded to P or LP if they were predicted to have this effect based on InterVar. Variant coordinates are based on Genome Reference Consortium Human Build 38 (GRCh38).

[b] This variant is not in ClinVar and it is classified as LP based on InterVar criteria.

[c] The ClinVar classification for this variant is VUS. This variant is classified as LP based on InterVar criteria.

[d] This variant is not in ClinVar and it is classified as P based on InterVar criteria.
